# Microalgae as a Sustainable Source of Antioxidants in Animal Nutrition, Health and Livestock Development

**DOI:** 10.3390/antiox12101882

**Published:** 2023-10-19

**Authors:** Alexandros Mavrommatis, Eleni Tsiplakou, Anastasia Zerva, Panagiota D. Pantiora, Nikolaos D. Georgakis, Georgia P. Tsintzou, Panagiotis Madesis, Nikolaos E. Labrou

**Affiliations:** 1 Laboratory of Nutritional Physiology and Feeding, Department of Animal Science, School of Animal Biosciences, Agricultural University of Athens, 75 Iera Odos Str., GR-11855 Athens, Greece; mavrommatis@aua.gr; 2 Laboratory of Enzyme Technology, Department of Biotechnology, School of Applied Biology and Biotechnology, Agricultural University of Athens, 75 Iera Odos Str., GR-11855 Athens, Greece; anazer@aua.gr (A.Z.); pantiora@aua.gr (P.D.P.); n.georgakis@aua.gr (N.D.G.); 3 Laboratory of Molecular Biology of Plants, School of Agricultural Sciences, University of Thessaly, GR-38221 Volos, Greece; gtsintzou@uth.gr (G.P.T.); pmadesis@certh.gr (P.M.); 4 Institute of Applied Biosciences, CERTH, 6th km Charilaou-Thermis Road, P.O. Box 361, Thermi, GR-57001 Thessaloniki, Greece

**Keywords:** animal nutrition, antioxidants, livestock, microalgae, monogastric diet, sustainability

## Abstract

Microalgae are a renewable and sustainable source of bioactive compounds, such as essential amino acids, polyunsaturated fatty acids, and antioxidant compounds, that have been documented to have beneficial effects on nutrition and health. Among these natural products, the demand for natural antioxidants, as an alternative to synthetic antioxidants, has increased. The antioxidant activity of microalgae significantly varies between species and depends on growth conditions. In the last decade, microalgae have been explored in livestock animals as feed additives with the aim of improving both animals’ health and performance as well as product quality and the environmental impact of livestock. These findings are highly dependent on the composition of microalgae strain and their amount in the diet. The use of carbohydrate-active enzymes can increase nutrient bioavailability as a consequence of recalcitrant microalgae cell wall degradation, making it a promising strategy for monogastric nutrition for improving livestock productivity. The use of microalgae as an alternative to conventional feedstuffs is becoming increasingly important due to food–feed competition, land degradation, water deprivation, and climate change. However, the cost-effective production and use of microalgae is a major challenge in the near future, and their cultivation technology should be improved by reducing production costs, thus increasing profitability.

## 1. Introduction

Over the past ten years, the feed, food, cosmetic, and nutraceutical industries have all become interested in the natural compounds from microalgae, due to the rising demand for natural antioxidants as an alternative to synthetic antioxidants [1,2]. Microalgae are an underutilized resource with the potential to produce antioxidants and novel bioactive molecules. They are attractive for developing biotechnological applications due to their high growth rate, simplicity in cultivation, production scalability, potential for genetic modification, low maintenance costs, and metabolic plasticity that can be directed and manipulated to produce target compounds by altering culture conditions [3,4]. Due to all these factors, the demand for algal biomass is expected to increase significantly in the coming years. It has been estimated that the global algae market will reach a value of USD 6.3 billion by 2028, up from USD 4.5 billion in 2021 [5].

From Ancient Greece to Icelandic sagas and all over the world, ample evidence suggests that both wild and domesticated animals approached coastal areas to feed on seaweed. After the intensification of livestock production systems and the consideration of current emerging issues such as food–feed competition and the hard-to-find arable lands for feedstuff production, the usage of algae was redefined. Corn and soybean, the two main conventional feedstuffs for animal feeding, are unsustainable, and therefore alternatives to these ingredients are required to maintain livestock performance, especially considering the latest exponential demands for animal protein [6,7]. Land degradation, water deprivation, and drastic changes in climate are also significant challenges for the future of the livestock sector signifying the need to explore highly sustainable alternatives to conventional feedstuffs, which are not affected by environmental conditions. The cultivation of microalgae does not require rainfall, which would decrease competition with areas more suited for agricultural production or biodiversity conservation [8]. Although until recently, the use of algae as an alternative to conventional feedstuffs was advocated as a panacea for the aforementioned challenges, when applied in animal diets, their assessment unveiled two significant drawbacks. The high production cost of algae limits their inclusion levels in animal diets since this cost cannot be remunerated by the price of animal products [7]. Additionally, their cell walls inhibit nutrient release in the duodenum of monogastric animals and decrease their digestibility. Considering these issues, in the last decade, seaweeds and microalgae have been explored in animal nutrition as feed additives (in low inclusion levels) rich in bioactive compounds for livestock animals and aquaculture with the aim of improving both animals’ health and performance as well as product quality and livestock environmental impact. 

This review article aims to cover recent research progress on the antioxidant molecules in microalgae and their role as feed additives for improving both animals’ health and performance as well as product quality and livestock environmental impact.

## 2. Microalgal Diversity in Industrial Setting

Microalgae are a large group of organisms that are extremely diverse and heterogeneous from evolutionary and ecological viewpoints. Microalgal biomass is an excellent source of diverse bioactive compounds such as lipids, polysaccharides, carotenoids, vitamins, phenolics, and phycobiliproteins [1,2]. The bioactive properties of different industrially produced microalgae vary as a consequence of their physiology and biochemistry. The diversity of microalgal species gives rise to various antioxidant molecules, which makes microalgae the richest natural resource for nutritional and bioactive components [9,10,11]. The list of industrially produced microalgae includes genera spanning different classes, namely Chlorodendrophyceae (*Tetraselmis chui*, *Tetraselmis striata* CTP4); Chlorophyceae (*Haematococcus lacustris*, formerly *Haematococcus pluvialis*); Coccolithophyceae (*Tisochrysis lutea*); Bacillariophyceae (*Phaeodactylum tricornutum*, *Skeletonema* sp.); Eustigmatophyceae (*Nannochloropsis* sp.); Porphyridiophyceae (*Porphyridium* sp.); and Cyanophyceae (*Spirulina*). Among them, there are only a few microalgae that have “Generally Recognized as Safe” (GRAS) status as recognized by the FDA. These microalgae include *Arthrospira platensis* (*Spirulina*, *Cyanophyceae*), *Chlamydomonas reinhardtii*, *Auxenochlorella protothecoides* (Trebouxiophyceae), *Chlorella vulgaris*, *Dunaliella salina* (formerly *Dunaliella bardawil*) (Chlorophyceae), and *Euglena gracilis* (Euglenophyceae).

## 3. Antioxidant Compounds in Microalgae

**Figure 1:**
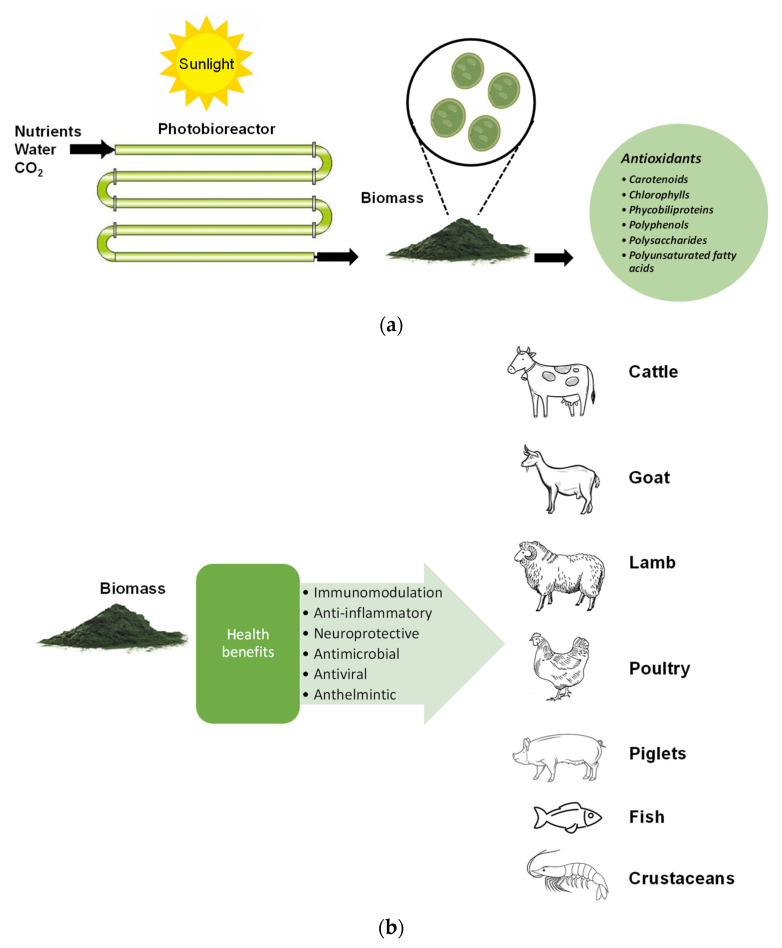
(**a**) Main antioxidant compounds produced by microalgae; (**b**) the beneficial properties of microalgae in animal health.

Microalgae live in habitats under high solar irradiation and as a consequence have a wide range of antioxidant compounds, which protect them from radiation and oxidation damage. High-value antioxidant compounds produced by microalgae include polyunsaturated fatty acids (PUFAs); carotenoids, including astaxanthin and lutein; chlorophylls; phycobiliproteins; and phenolic compounds [1,2,9,10,11,12,13] (Figure 1a). These compounds have immunomodulation, anti-inflammatory, neuroprotective, antimicrobial, antiviral, and anthelmintic properties, beneficial to animal health [14] (Figure 1b). Antioxidants help to prevent oxidative damage, which mostly occurs from oxygen’s reduced states. There are many types of reactive oxygen species [15]. The antioxidant function can be derived from two sources: the activity of antioxidant enzymes [12,13] or the production of molecules that serve as sacrificial scavengers of reactive oxygen species [2]. Additionally, antioxidant activity can be divided into two major mechanisms of action: limiting reactive oxygen species in the digestive tract to lessen oxidative stress on the gut microbiome and epithelial cells or transporting antioxidants into the circulation for distribution throughout the body. There are still knowledge gaps about the effectiveness of the antioxidant qualities of microalgal meals at all levels, from species differentiation to effects on the gut microbiota and movement through the gut lumen to their effects on animal physiology. This will be useful for further research in the next decade.

### 3.1. Polyunsaturated Fatty Acids (PUFAs)

Polyunsaturated fatty acids (PUFAs) include docosahexaenoic acid (DHA), eicosapentaenoic acid (EPA), arachidonic acid (AA), and γ-linolenic acid (GLA), and are well known for being advantageous to both animal and human health [16,17,18]. Several microalgal species have been recorded as sources of PUFAs, such as *Phaeodactylum tricornutum*; *Monodopsis subterranea* (formerly *Monodus subterranea*) (Xanthophyceae); *Porphyridium purpureum* (formerly *Porphyridium cruentum*) (Porphyridiophyceae); *Chaetoceros calcitrans* (Mediophyceae); *Nannochloropsis* sp. (Eustigmatophyceae); *Crypthecodinium cohnii* (Dinophyceae); *Isochrysis galbana* (Coccolithophyceae); and *Rebecca salina* (formerly *Pavlova salina*) (Pavlovophyceae) [17,18,19,20]. Among the physiologically important fatty acids, docosahexaenoic acid is the most sensitive to oxidation, whereas palmitic acid is the least oxidizable. The oxidation stability of fatty acids is directly connected with the degree of unsaturation.

### 3.2. Carotenoids

Carotenoids are abundant in microalgae [21,22]. These colorful compounds are well known for their antioxidant properties [14,23,24,25]. They are used as natural colorants [26,27,28] and as food and feed additives or health supplements. They are divided into two groups: carotenes and xanthophylls. The former group of compounds are oxygen-free hydrocarbons, such as α-carotene and β-carotene, while the latter compounds are oxygenated derivatives of carotenes (including lutein, violaxanthin, zeaxanthin, fucoxanthin, and astaxanthin) [25,29]. The main sources of carotenoids are microalgae belonging to the Chlorophyceae. Many microalgae accumulate carotenoids but the dominant species extensively studied are *Dunaliella salina*; *Haematococcus lacustris* (Chlorophyceae); *Chromochloris zofingiensis* (formerly *Chlorella zofingiensis*) (Trebouxiophyceae); and *Chlorella vulgaris* (Chlrophyceae), especially due to the capability of commercial production in large-scale cultures [30,31]. These microalgae may generate a variety of pigments, including carotenes (β-carotene and lycopene) and xanthophylls (astaxanthin, violaxanthin, antheraxanthin, zeaxanthin, neoxanthin, and lutein). Other microalgae phyla generate other compounds such as fucoxanthin, diatoxanthin, and diadinoxanthin [23]. Currently, the two pigments with the highest global market are β-carotene and astaxanthin from the genera *Dunaliella* and *Haematococcus*, respectively [23,32,33]. More specifically, β-carotene is commonly produced by *Tetradesmus almeriensis* (formerly *Scenedesmus almeriensis*), *Dunaliella salina* (formerly *Dunaliella bardawil*), and *Dunaliella tertiolecta* (Chlorophyceae) [14,19,20,23,34,35,36]; however, the best source for its production is *Dunaliella salina* [33,37]. 

Astaxanthin, a red xanthophyll pigment, is the second most widely used carotenoid [38]. It displays an efficacious antioxidant activity [39,40,41] and shows about ten times higher antioxidant activity than other carotenoids [19,23,34,35]. It is produced by several microalgae such as *Chlorella zofingiensis*, *Chlorococcum* sp., and *Scenedesmus* sp., as well as the yeast *Xanthophyllomyces dendrorhous* [14,20,24]. Interestingly, the microalga *Haematococcus lacustris* [35,42], under certain cultivation conditions, can accumulate up to 7% astaxanthin on a dry weight basis [37]. Therefore, *Haematococcus lacustris* is seen as the most favorable species for industrial scale production of natural astaxanthin [33,43]. Astaxanthin is widely used in aquaculture feed as a dye agent for fish and shellfish flesh due to its red color [14,35,42]. It is also exploited as an antioxidant supplement to improve the health and production performance of broiler chicken [44]. 

Lutein is another important carotenoid [45]. It is used for the pigmentation of animal tissues and products [46]. It also shows bioactive beneficial properties in chronic diseases, such as cataracts, atherosclerosis, blindness, or decreased vision [47,48,49]. Lutein is produced by several microalgae such as *Chlorella* sp. [50,51], *Muriellopsis* sp. [52], *Scenedesmus* sp. [53], and *Chlamydomonas* sp. [54]. It is widely used for the natural coloration of foods, drugs, and cosmetics [55]. Lutein-producing strains include *Muriellopsis* sp., *Auxenochlorella protothecoides* (formerly *Chlorella protothecoides*) (Trebouxiophyceae), *Chromochloris zofingiensis* (formerly *Chlorella zofingiensis*), *Pleurastrum insigne* (formerly *Chlorococcum citriforme*), *Neospongiococcum gelatinosum*, and *Tetradesmus almeriensis* (Chlorophyceae) [14,20,56].

Other important carotenoid pigments with high commercial value include lycopene, violaxanthin, and zeaxanthin [57]. Lycopene is extensively used in cosmetic formulations as a sunscreen and antiaging compound [20,34]. It also displays anticarcinogenic and antiatherogenic properties [20]. Violaxanthin, an orange carotenoid pigment, is well known for its anti-inflammatory and anticancer properties. It is produced by *Chloroidium ellipsoideum* (formerly *Chlorella ellipsoidea*) (Trebouxiophyceae) and *Dunaliella tertiolecta* [19,23] strains. Zeaxanthin is a yellow carotenoid that has found successful applications in the pharmaceutical, cosmetic, and food industries. The industrial production of zeaxanthin is mainly achieved by *Tetradesmus almeriensis* and *Nannochloropsis oculata* [20,35]. Other noteworthy carotenoids include canthaxanthin, β-cryptoxanthin, and fucoxanthin, which have shown significant tanning, anti-inflammatory, and anticancer properties, respectively [21,22,34,58,59], and therefore they have been used in the pharmaceutical or cosmetic industries.

### 3.3. Chlorophylls 

Chlorophylls are found in all photosynthetic microalgae [60,61]. Due to their green pigmentation, they are becoming increasingly important as colorants in the food industry as well as in the pharmaceutical and cosmetic industries [14,21,24,57]. Chlorophyll a and chlorophyll b also occur in the form of sodium and copper derivatives. The latter types of derivatives are mainly used as food additives or in drinks [14]. Microalgae that belong to the genus *Chlorella* contain chlorophyll to about 7% of their biomass, five times more than that of *Arthrospira* [36,61].

### 3.4. Phycobiliproteins 

Phycobiliproteins are only found in cyanobacteria and some red algae [62,63,64]. Phycobiliprotein, a high-potential molecule, has been utilized commercially as a natural dye and has a variety of applications in the pharmaceutical industry [63,64,65]. Due to their powerful and highly sensitive fluorescent properties, they are used as markers for certain immunological methods, such as flow cytometry, microscopy, and DNA tests [14,24,32,35,57,63,64,66]. On an industrial scale, these pigments are produced from the species *Porphyridium* sp., *Arthrospira* sp., and *Aphanizomenon flosaquae* (Cyanophyceae) [14,19,20,24,34,35].

**Figure 2:**
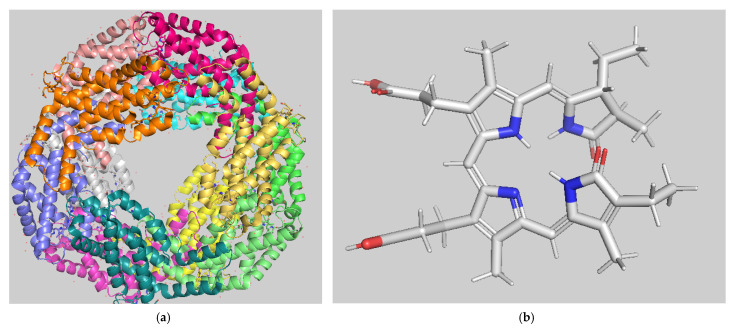
(**a**) The structure of C-phycocyanin from *Arthrospira platensis* (formerly *Spirulina platensis*) at 2.2 Å resolution (PDB accession number 1GH0); (**b**) the structure of phycocyanobilin (PCB), a linear tetrapyrrole chromophore covalently attached to protein subunits of C-phycocyanin.

The predominant pigment in the phycobiliprotein family is phycocyanin [63,64]. Phycocyanin is a blue protein in cyanobacteria, rhodophytes, and cryptophytes that possesses the blue tetrapyrrole chromophore, phycocyanobilin, with fluorescent and bioactive properties (Figure 2). Phycocyanin is a water-soluble, non-toxic, and blue-colored photosynthetic pigment that have been used in food, cosmetic, and pharmaceutical industries. Over the years, the biological function of phycocyanin has been extensively studied. For example, numerous studies have investigated its antioxidative, anti-inflammatory, anticancer, and antimicrobial activity, as well as its effects on neurodegeneration, diabetes, wound healing, and hyperpigmentation [67,68,69]. Extensive studies during the last two decades concerning purification, biochemical, and structural properties has resulted in a detailed description of phycocyanin’s architecture, structure, and bioactivity [67,70]. The primary biotechnological potential of phycocyanin seems to be its application as a natural pigment, replacing toxic synthetic dyes. However, a growing number of evidences have demonstrated that phycocyanin also exhibits bioactive properties related to health benefits such as antiaging, antioxidant, anticancer, neuroprotective, and anti-inflammatory activities [70].

### 3.5. Polysaccharides 

Polysaccharides are polymers consisting of saccharide units linked with glycosidic bonds attached to the cell wall or released into the medium (exopolysaccharides) [71]. Polysaccharides isolated from a range of microalgal species (e.g., *Arthrospira platensis*, *Porphyridium purpureum*, *Dunaliella salina*, *Dixoniella grisea* [formerly *Rhodella reticulata*) (Rhodellophyceae), and *Schizochytrium* sp.] exhibit in vitro antioxidant properties and ability to effectively scavenge superoxide radicals, hydroxyl radicals, and hydroxyl peroxides [2,43,71,72]. The diverse biological activities of polysaccharides in microalgae are due to their complex structural features, including molecular weight, composition of sugar residues, types of glycosidic linkages, nature of monosaccharides, and the presence of some sugar-free units (sulfate, methyl, organic acids, amino acids, or amines) in the main skeleton of polysaccharides [2,71,72].

### 3.6. Polyphenols 

Polyphenols are a large group of secondary metabolites comprising phenolic acids, flavonoids, isoflavonoids, stilbenes, lignans, and phenolic polymers [71,72,73,74]. Phenolic compounds, especially flavonoids and phenolic acids, exhibit high antioxidant function. The extraction and purification of polyphenols from microalgae is challenging, and improvements in analytical methodology are needed to facilitate more detailed characterization of their structure and function. Recently, a study was carried out on polyphenols extracted from two different microalgal species: *Nannochloropsis* sp. and *Arthrospira*/*Spirulina* sp. [75].

## 4. Antioxidants and Gut Microbiota

Several studies have investigated the relationship between antioxidants and the gut microbiota in animals [76,77,78,79,80,81]. Some studies have suggested that the antioxidants present in microalgae can influence the composition of the gut microbiota [82]. For example, dietary antioxidants like polyphenols and flavonoids found in fruits and vegetables have been shown to promote the growth of beneficial bacteria like *Bifidobacterium* and *Lactobacillus* while reducing the abundance of potentially harmful bacteria [79,82,83,84,85]. Some antioxidants, especially dietary fibers and polyphenols, can serve as substrates for bacterial fermentation in the gut. This fermentation process produces short-chain fatty acids (SCFAs), which have beneficial effects on gut health. SCFAs serve as an energy source for gut cells, regulate inflammation, and support the growth of beneficial bacteria [84,85]. Furthermore, antioxidants possess anti-inflammatory properties. Inflammation in the gut is associated with various gastrointestinal disorders. Therefore, by reducing inflammation, microalgae may help maintain a healthy gut environment and support the growth of beneficial gut bacteria [86,87,88].

Antioxidants may also contribute to the integrity and function of the gut barrier, which acts as a protective barrier between the gut microbiota and the rest of the body [89]. A healthy gut barrier prevents the translocation of harmful bacteria or their by-products into the bloodstream.

## 5. The Effect of Microalgae on Animal Performance

As mentioned before, the high production cost of microalgae due to current cultivation technology, together with their low digestibility in monogastric species, are the limiting factors for their inclusion in animals’ diets substituting conventional feedstuffs. For this reason, research has focused on lower dietary supplementation levels with the aim of improving animal performance by enhancing nutritional physiology pathways. Moreover, the chemical composition of microalgae (rich in protein or fat content) affects animal performance differently. It has been reported that the dietary inclusion of high-protein species improved the body condition and average daily gain (ADG) of dairy cows and lambs, respectively [90,91], while diets containing high-fat microalgae namely *Schizochytrium* spp. negatively affected lambs’ performance through a reduction in their dry matter intake (DMI) [92,93]. Mavrommatis and Tsiplakou (2020) [94] also observed a 30% reduction in DMI when *Schizochytrium* spp. included at 3% in goats’ diet. This reduction in DMI is usually attributed to the fish-like odor of microalgae or to the high-fat content of *Schizochytrium* spp., which might impair the hypophagic effect on the brain’s satiety center.

In broilers, the dietary inclusion of *Arthrospira* sp. in different levels (ranging from 0.5% to 21%) had no effects on performance parameters [95,96,97]. On the other hand, the low dietary inclusion of *Chlorella* sp. (0.00003% to 1%) consistently increased ADG and overall growth performance in broilers, possibly through beneficial cellular remodeling owing to microalgal secondary metabolites and bioactive compounds [98,99]. Thus, both nutrients’ complementarity and the digestibility of microalgae, especially in the higher inclusion levels, might explain these results. Moreover, the dietary inclusion of fat-rich microalgae *Schizochytrium* spp. (3.7–7.4%) increased broilers’ DMI and consequently their productive performance from 21 to 35 days old [100,101]. These findings indicate that not only the dietary inclusion level but also the chemical composition of microalgae needs to be taken into consideration in diet formulation. 

In pigs, negligible improvement in growth performance was observed when fed with diets containing *Arthrospira platensis* (0.2–2%) [102]. In fattening pigs, the inclusion of 0.2% *Arthrospira platensis* (*Spirulina*) significantly increased ADG without affecting back fat thickness [103]. However, the combined dietary inclusion of Spirulina with *Chlorella vulgaris* at 1% on weaned piglets for only 14 days did not affect ADG even though a potential effect on intestinal development through the regulation of a mild digestive disorder was reported [104]. Thus, it could be hypothesized that the trial interval of the previous study was limited in order for substantial changes in animal performances to be unveiled. No effect on ADG, final body weight, and carcass traits of female pigs was observed either when 0.1% *Chlorella* spp. was included in their diet [105]. The low supplementation level of microalgae might be the reason for the absence of any significant effect. Indeed, variable dietary inclusion levels of *Schizochytrium* spp. (1.10% to 5.51% from day 79 to 106 and 0.39% to 1.94% from day 107 to 120) increased ADG and FCR without affecting DMI [106], while lower inclusion levels of the same microalgae (0.25–0.50%) did not change the growth performance of finishing pigs [107]. The former experimental trials in pigs signify once again the importance of supplementation levels and the duration of administration.

## 6. The Effect of Microalgae on Animal Health

The biochemical profiles of microalgal species (*Spirulina*, *Chlorella* sp., *Nannochloropsis granulat*, *Schizochytrium*, and *Tetraselmis chui*) commonly used for formulating animal feed include essential amino acids and polyunsaturated fatty acids (PUFAs) such as eicosapentaenoic acid (EPA), docosapentaenoic acid (DPA), and docosahexaenoic acid (DHA) that are not synthesized in animals’ organism, as well as antioxidant compounds (such as carotenoids and flavonoids, trace elements, minerals, and vitamins). Extensive scientific evidence has been documented highlighting the impact of these molecules on animal health. However, microalgae also represent a source of unexploited bioactive compounds, which may have exceptional properties and significant applications, including, but not limited to, lipoproteins, sterols, and alkaloids [108]. 

Amongst the biomolecules present in microalgae, PUFAs have gained significant interest since these vital nutrients have been correlated with human health benefits [109]. The advantage of microalgae regarding PUFA formation and accumulation lies in an efficient elongase–desaturase network that is common in many algal species [110,111]. Indeed, long-chain polyunsaturated fatty acids (LCPUFAs) could regulate animals’ pro-inflammatory response induced by farming conditions, especially in high-yielding individuals, through the production of eicosanoids with much less pro-inflammatory power or by inhibiting pro-inflammatory cytokines, resulting in the suppression of low-grade inflammation and stress. In this light, in a recent study by Mavrommatis et al. (2021) [112], the dietary supplementation with 20, 40, and 60 g *Schizochytrium* spp. downregulated the transcriptional profile of the TLR4 pathway in goats’ blood monocytes. The former induced a cascade of downregulations in pro-inflammatory cytokines (*IFNG*, *IL1B*, *IL2*, *IL8*, and *TNF*) and chemokines (*CCL5* and *CXCL16*) in both blood monocytes and neutrophils. The mechanism that underlies this pro-inflammatory suppression may be attributed to the immunomodulatory effects of both DHA and ω6-DPA fatty acids contained in *Schizochytrium* biomass on immune cells’ intramembrane receptors such as Toll-like receptors (TLRs) and G protein-coupled receptor 120 (GPR120) [112]. Another possible mode of action may be related to the metabolism of LCPUFAs in immune cells and their utilization to synthesize immunomodulatory mediators (eicosanoids). To further investigate these speculations, Kyriakaki et al. (2023) [113] assessed the expression of genes involved in eicosanoid production in monocytes and neutrophils of goats fed with *Schizochytrium* spp. and found a significant decrease in the expression of genes that regulate both cyclooxygenase (*COX2*) and lipoxygenase (*5-LOX*) pathways, indicating an overall suppression of pro-inflammatory response. These lines of evidence may have significant implications for dairy ruminants’ resilience toward commercial farming since a low-grade pro-inflammatory response that activates the immune system can lead to severe competition for nutrient availability [114]. More specifically, animals’ energy and nutrient requirements for maintenance and production can often be overlooked with regard to their role in immune function demands. It has been estimated that the activation of the immune system requires as much as 10–30% of metabolizable energy [114]. These energetic demands are supported by studies that have reported the utilization of 1 kg of glucose (within a 12 h period) for the immune system’s activation in both cattle and swine [114]. Considering the topic of inflammation, Caroprese et al. (2012) [115] reported that a mixture of phytosterols from *Dunaliella tertiolecta* reduced the cytokine production in a sheep model of inflammation. Moreover, *Chlorella sorokiniana* suppressed the peripheral blood mononuclear cell proliferation and pro-inflammatory cytokine levels in sheep in vitro [116]. 

The bioactive compounds of microalgae possess several health benefits such as boosting the immune system, which will eventually reduce antibiotic dependence in livestock farming [108]. Indeed, Amaro et al. (2011) [117] summarized the potential antibacterial properties of microalgae, reporting that specific fatty acids, organic acids, and extra-metabolites could effectively counteract MRSA, *E. coli*, *Vibrio*, *Salmonella*, *Pseudomonas*, etc. Fries-Craft et al. (2021) [118] assessed the inclusion of 0.175% of a mixture consisting of Spirulina and *Chlorella* in the broilers’ diet. Microalgae maintained intestinal integrity during the coccidiosis challenge and protected jejunal villus height. During the *Eimeria* challenge, splenic T cells in microalgae-fed broilers did not provide evidence of recruitment to peripheral tissues. These outcomes suggest that the ingredients in microalgae modified the immune response in a manner that reduced recruitment from secondary lymphoid organs in addition to protecting intestinal physiology. Dietary supplementation with 1% Spirulina or *Chlorella* microalgae as an alternative to antibiotic use was studied by Furbeyre et al. (2017) [104]. Diarrhea incidence was reduced in *Chlorella*-fed pigs compared with the control, Spirulina, and antibiotic (colistin) groups. Villus height at the jejunum was greater in microalgae-fed pigs compared with the control and antibiotic-fed pigs. This study reported a potential effect of both Spirulina and *Chlorella* supplementation on intestinal development and further advantages of *Chlorella* supplementation to manage mild digestive disorders. Similarly, the inclusion of 0.1% fermented *Chlorella* in growing pigs’ diet improved growth performance, nutrient digestibility, and fecal microbial structure (higher *Lactobacillus* and lower *E. coli*), and decreased fecal noxious gas emission [119]. These sets of evidence demonstrate that microalgae and their derivatives could be useful ingredients in animal feed for minimizing the dependence on antibiotics, aiming to control antibiotic resistance and support animal health using natural bioactive compounds.

Besides its antibacterial, antiviral, and anthelmintic properties, microalgal biomass can be considered as a multi-component antioxidant system that in general is more effective through the interactions between the different antioxidant components. In this context, El-Bahr et al. (2020) [120] observed lower values of malondialdehydes (MDAs) and protein carbonyls (PCs) in the breast muscle of broilers, while the activity of superoxide dismutase (SOD) was increased due to dietary supplementation with microalgal species (*Arthrospira platensis*, *Chlorella vulgaris*, and *Halamphora coffeiformis* (formerly *Amphora coffeiformis*)). Remarkably, the dietary supplementation with microalgae-based antioxidants minimized the detrimental effect of mycotoxin-contaminated feed and partially improved the feed conversion ratio (FCR) in both heat-stressed and unstressed broiler chickens [121]. A recent study conducted by Christodoulou et al. (2022) [122] reported that the dietary supplementation with Spirulina significantly increased the antioxidant defense of sheep organisms through the higher activity of SOD, catalase (CAT), and glutathione peroxidase (GSH-Px), while the protein oxidative index (PC) was decreased. Interestingly, Spirulina used in the former study was derived through secondary sorting (no competition with the food and cosmetic industry), thus opening new valorization directions in the feed industry.

Even though microalgae and their bioactive compounds have been associated with many beneficial properties on animal health, the first principle of toxicology is that “all things are poisonous, and it is the dose that distinguishes between a drug and a poison” (Paracelsus C15th). More specifically, in an effort to fortify animal products with PUFAs through the supplementation of microalgae, the high propensity of PUFAs to oxidation could lead to a severe immune-oxidative burst. Dietary PUFA overload can activate cellular superoxide anion generators like xanthine and NADPH oxidases, resulting in superoxide anion formation and launching a cascade of pro-oxidant incidences [123]. Indeed, the high supplementation levels of *Schizochytrium* spp. (40 and 60 g/day) in goats’ diet increased the activity of NADPH oxidase in blood plasma [124] and the mRNA levels of *NOX1* and *NOX2* in their monocytes and neutrophils [113], while a lower supplementation level (20 g) did not. Similarly, the dietary inclusion of *Schizochytrium* spp. significantly impaired sheep’s oxidative system as reflected by the higher levels of MDAs and PCs. Another risk of incorporating microalgae into the human food chain is related to their ability to accumulate toxic metals. In semi-arid regions where the availability of fresh water is scarce, water wastes such as those obtained by mines are frequently used. Therefore, careful attention must be given before use as animal feed [125].

## 7. The Effect of Microalgae on Animal Product Quality

Even if the current cultivation technology does not allow for the substantial substitution of conventional feedstuffs by microalgae, and their inclusion in low levels in animal diets does not significantly improve animal performance, their application as feed additives for the promotion of animal product quality has been well documented with promising outcomes [110,126]. 

Laying hens fed with diets supplemented with *Microchloropsis gaditana* (formerly *Nannochloropsis gaditana*), containing ω-3 LCPUFAs such as EPA and DHA, resulted in the accumulation of these ω-3 FAs in the egg yolk [127]. Interestingly, a higher proportion of DHA than EPA accumulated in eggs when *Nannochloropsis oculata* was fed to laying hens, even though this microalga is richer in EPA [108]. In addition to the enrichment of eggs with beneficial LCPUFAs, the inclusion of 20% *Nannochloropsis oculata* in laying hens’ diet increased the lutein and zeaxanthin content to 1.3 mg/egg [128]. In this context, the accumulation of carotenoids in egg yolk results in a darker orange color, which increases consumer acceptance [129]. Another important aspect of bioactive compounds in microalgae is related to their bioavailability. More specifically, it has been reported that lutein contained in *Chlorella* is incorporated more efficiently in eggs than synthetic carotenoids, also resulting in improved oxidative stability of yolk lipids [130]. Nevertheless, it should be mentioned that high levels of LCPUFAs in hens’ diet can decrease tocopherol availability for proper egg yolk formation and induce pro-oxidant incidences with a further impact on birds’ health and homeostasis [131]. Hence, the importance of supplementation levels should not be overlooked. 

Considering the effect of dietary supplementation with microalgae on poultry and pigs’ meat quality, the study of Martins et al. (2022) [126] has comprehensively summarized the latest insights. The dietary inclusion of Spirulina (4% or 8%) increased the yellow appearance of broilers’ muscles, skin, fat, and liver, which increased the commercial value of the meat and consequently the consumers’ acceptance [132]. Similarly, a high inclusion level (10%) of *Chlorella vulgaris* increased tenderness, yellowness, and total carotenoids in the breast and thigh meat of broilers [133]. Additionally, the dietary inclusion of *Schizochytrium* spp. rich in DHA increased the ω-3 content in the breast and thigh of broilers in numerous studies investigating a wide range of levels from 0.1% to 7.4% [100,101,119,134,135,136,137]. However, it should be highlighted that the higher dietary supplementation levels of *Schizochytrium* spp. also increased the concentration of lipid peroxidation metabolites [100,101]. In terms of pigs’ meat quality, the effects of *Schizochytrium* spp. are quite similar to those observed in poultry [126]. Moreover, regarding the supplementation of protein-rich microalgae, the inclusion of *Chlorella vulgaris* at 5% increased the total carotenoid content in meat in weaned piglets and grower pigs [126,138].

In ruminants, the concept of enriching meat and milk with marine-origin fatty acids is more complicated. Due to the symbiotic microbiome colonizing the rumen, about 70–100% of PUFAs present in the feed are biohydrogenated, resulting in the formation of saturated fatty acids, mainly stearic acid, which is transferred to tissues and milk. However, there is another ruminal biochemical procedure that can be manipulated through dietary marine fatty acids, aiming to enrich products with PUFAs. The increased flow of LCPUFAs into the rumen changes biohydrogenation pathways, resulting in the accumulation of vaccenic acid due to the procedure’s incompleteness. Vaccenic acid is desaturated through the activity of Δ9 desaturase to conjugated linoleic acid (CLA), a fatty acid with significant health benefits that humans receive through the consumption of milk and meat [139]. Thus, although the transfer efficiency of EPA, DPA, and DHA is quite low in ruminants due to their biohydrogenation [94,140], another important biomolecule can be formed. In this context, dietary supplementation with *Schizochytrium* spp., rich in ω6-DPA and DHA, enriched ovine [141] and caprine [94,142] milk with DHA, ω6-DPA, and CLA resulting in a two-fold increase in total milk PUFA content. More specifically, microalgae-fed goats and sheep produced milk fortified with up to four- and six-fold increased proportions of CLA, respectively. Additionally, in both goat and sheep milk, the ω6/ω3 ratio, the health-promoting index, and the atherogenic index were significantly improved, setting new horizons for the development of functional dairy products enriched with beneficial fatty acids for human health. The former constitutes an important aspect of the industry since ruminants’ milk has been criticized for its high proportion of saturated fatty acids, which have been correlated with a high risk for human cardiovascular diseases [139].

On the contrary, the high accumulation of PUFAs in milk and dairy products increases their propensity to oxidation [124]. Indeed, the high inclusion level of *Schizochytrium* spp. in goats’ diet impaired milk oxidative status through the accumulation of toxic aldehydes such as MDAs and protein oxidation products (PCs) [124,142]. Dairy sheep were found to be more prone to oxidation since MDAs in milk were increased even in the lowest supplementation level [141]. Nevertheless, Christodoulou et al. (2022) [122] reported that Spirulina supplementation in dairy sheep diet significantly increased the activity of antioxidant enzymes, namely SOD, CAT, and GSH-Px, in milk and its total antioxidant capacity. The former constitutes an important aspect since raw milk is frequently oxidized during its transportation to the industry; thus, receiving milk with a more stabilized oxidative status can improve its overall life span. The holistic consideration of the abovementioned trials allows us to highlight the potential benefits of combining microalgae rich in PUFAs and antioxidant compounds simultaneously, formulating feed additives aiming to fortify milk and dairy products with beneficial fatty acids for human health while concurrently controlling any side effects related to PUFAs’ oxidation.

## 8. Microalgae in Monogastric Diets: The Use of Carbohydrate-Active Enzymes

The digestibility of microalgal biomass can be impaired by the complicated cellulosic cell walls of most microalgal species, distantly related to the architecture of plant cell walls. Little is known of the exact nature of the cell walls of microalgae in general. Most relevant studies are conducted in commonly used model organisms, such as *Chlorella* [143,144] and *Nanochloropsis* [145], but they are often contradictory, due to the significant variety observed depending on the species, the growth stage, and often the cultivation medium [146]. A relatively simple and straightforward method to determine the composition of algal cell walls is the hydrolysis of the material in harsh conditions and the compositional analysis of the resulting monomers (sugars or amino acids for example), as an indirect way to predict the polymers present and their proportions. Using this approach, Spain and Funk (2022) [143] characterized the composition of several Nordic species of microalgae, including *Chlorella vulgaris*, *Scenedesmus* sp., *Haematococcus lacustris*, and *Coelastrella* sp., and their changes according to growth phase. While for all strains, the same monosaccharides were present (arabinose, rhamnose, fucose, xylose, mannose, galactose, glucose, galacturonic, and glucuronic acids), the glucose content was found to considerably vary for *Coelastrella* sp. and *Scenedesmus* sp. in different growth phases. Accordingly, glycine, glutamic acid, aspartic acid, threonine, and alanine were found to be the most abundant amino acids in all strains, but the proportion between polar and non-polar amino acids shifted throughout the course of cultivation for *Scenedesmus* sp. Moreover, the protein-to-carbohydrate ratio also shifted during growth for all strains. Similarly, Weber et al. (2022) [144] studied the cell wall composition of *C. vulgaris* using alkaline or acidic extraction. Alkali extracts mainly contained glucosamine, indicating the presence of a chitin-like polymer, while acidic extracts mainly consisted of glucose, indicating the presence of cellulose or starch. Galactose, mannose, rhamnose, and uronic acids were also present, indicating the presence of pectin- and galactan-like polysaccharides, together with glycoproteins. 

Nonetheless, most studies confirm that microalgal cells are enveloped in a thick and recalcitrant cell wall, containing various carbohydrate polymers, such as pectin, chitin, cellulose, β-glucan, β-galactan, mannan, and other hemicelluloses, as well as hydroxyproline-rich glycoproteins, but most importantly algaenan, a highly recalcitrant aliphatic lignin-like polymer consisting of long mono- or di-unsaturated fatty acids, connected with ester and ether bonds and substituted with amide and pyrrole groups [147]. Algaenan is considered an indigestible polymer and a major constituent of organic matter sediments in soil and marine environments [145].

Due to the complexity of the material, efficient treatment methodologies must be developed. Mechanical treatments have been previously applied, but the energy cost and the almost complete destruction of the algal cells are significant drawbacks of such methods, prohibiting their industrial use [148]. Enzyme treatment seems to be the optimal approach to improve the digestibility of algal biomass since it is an environmentally friendly alternative, it does not require the use of organic solvents, and most enzymes used in feed production are approved for animal consumption. To this end, there is a significant number of experimental studies targeting the formulation of optimal enzyme mixtures for increasing the digestibility of microalgae, but due to the complexity and heterogeneity of the material, the results are often contradictory. For example, Gerken et al. (2013) [149] studied the viability of microalgal cells after different enzymatic treatments, in *Nannochloropsis*, *Nannochloris*, and *Chlorella* strains. Their results revealed that no single enzyme, except lysozyme to a certain extent, impaired the viability of all the tested microalgae, but the application of enzyme mixtures achieved this effect. For *Chlorella*, the combination of lysozyme and sulfatase or trypsin resulted in almost complete cell permeability. However, chitinase, chitosanase, cellulase, pectinase, and phospholipase also induced an altered morphology of the cell wall. The necessity for combined enzyme action for the efficient extraction of fatty acids was also evident in the work of Liang et al. (2012) [150], where among the various proteases tested, as well as cellulose-acting enzymes, the best combination was found to be trypsin together with snailase, an enzyme mixture containing cellulase, hemicellulase, pectinase, and β-glucuronidase. The group of Zuorro et al. also studied the extraction of fatty acids from *C. sorokiniana*, testing commercial enzyme preparations including cellulase, pectinase, lysozyme, and hemicellulases [151]. The optimal enzyme mixture contained β-1,4-xylanase and β-1,4-mannanase, resulting in over 70% lipid recovery, highlighting the necessity of complementary enzyme specificities working in synergy to achieve optimal results. The disruption of algal cell walls of *C. zofingiensis* was studied using crude enzyme mixtures from several bacterial strains grown in wheat bran as an enzyme inducer [152]. The crude enzyme extracts were found to contain cellulase, xylanase, and laccase activities, and they significantly disrupted the algal cell wall, resulting in increased reducing sugars in the supernatant, as well as lipid extraction efficiency. 

Regarding the enzymatic digestion of *Chlorella* cell walls, the group of Coelho et al. tested more than 200 carbohydrate-acting enzymes and sulfatases on *C. vulgaris* biomass, revealing 29 of them with a certain degree of activity [153]. The most effective candidates, including an exo-β-glucosaminidase, an alginate lyase, a peptidoglycan deacetylase, and a lysozyme, were tested as ternary mixtures for the optimization of reducing sugar release, leading up to 8-fold higher release of reducing sugars and 23-fold higher protein release, while the release of fatty acids was marginally improved. The same group used this approach to study the degradation of the cell walls of the microalga *Arthrospira platensis*, resulting in an efficient enzyme mixture containing only two enzymes, lysozyme and α-amylase [154]. The two-enzyme mixture resulted in 7-fold higher reducing sugars, 1.15-fold higher release in chlorophyl α, while the release of fatty acids was also facilitated. 

*Nannochloropsis* is another microalgal species with significant potential as animal feed. The group of Lavecchia et al. have studied the enzymatic pretreatment of the biomass from this species in detail. The tested enzymes included cellulase, mannanase, glucanase, galactanase, xylanase, esterase, and lysozyme, and they all resulted in increased lipid recovery. The most effective enzymes were found to be cellulase, mannanase, glucanase, and galactanase, which were further studied in binary and ternary combinations in order to design an efficient enzyme cocktail for this strain, resulting in a maximum of 37.2 g of lipids per 100 g of biomass [155]. In a follow-up study, the same authors achieved over 70% of lipid extraction yield from the same strain, with the synergistic effect of cellulase and mannanase. Moreover, they showed that the crystallinity of cellulose was increased in the cell walls, indicating the degradation of amorphous cellulose [156]. 

Overall, it is evident that the cost-effective treatment of microalgal biomass for improving its digestibility can be ideally implemented with combinations of enzymes with different specificities, targeting the various constituents of the cell wall. However, significant research effort is required for the design of tailored enzymatic cocktails depending on the available material, since the heterogeneity of the microalgal biomass hinders the application of universally efficient enzyme formulations.

## 9. Environmental Aspects of Using Microalgae in Animal Nutrition 

The Paris Agreement’s aim of limiting the increase in global temperature to 1.5 °C above preindustrial levels demands rapid and ambitious mitigation strategies aiming to reduce global greenhouse gas (GHG) emissions while simultaneously attaining a significant reduction in the amount of methane (CH_4_) produced by the agricultural sector [157]. Ruminants produce a significant amount of methane emitted through eructation as a normal biochemical function for the neutralization of CO_2_ and H_2_ formed in the rumen due to the microbial fermentation of the feed. 

Green, brown, and red seaweeds are key marine habitats rich in bioactive compounds such as bromoform (CHBr_3_), which inhibits methanogenesis. It is believed that CHBr_3_, along with other halogenated volatile organic compounds (VOCs), competitively bind to the enzymes and reductases that facilitate the final steps of reducing CO_2_ and H_2_ by methanogens (Archaea) into CH_4_. Bromoform is found within many seaweed species in low concentrations but has been found to accumulate in higher levels in the red seaweed *Asparagopsis taxiformis* [158]. Indeed, the inclusion of *A. taxiformis* (0.25% of organic matter (OM)) in beef diets reduced methane emission by 51 g/kg DMI, while a higher inclusion level (0.50% of OM) further reduced methane by 75 g/kg DMI [159]. Although *A. taxiformis* constitutes an important ally of ruminants against their high criticism as environmental polluters, dietary supplementation with this seaweed is negatively associated with one-health concept concerns mainly due to its bromoform content, a compound with potential carcinogenic properties.

Bromoform has been associated with ongoing health and environmental concerns even when included at low doses: The daily consumption of 67 g *A. taxiformis* (84.42 µg bromoform) resulted in rumenitis and residues in both urine and milk (10 and 9.1 µg bromoform, respectively) of cows [160]. The health and residue issues of bromoform should be considered with caution, as there are still very few studies published that define the long-term effects of feeding bromoform-rich seaweed on animal productivity, animal health, and residue deposition in milk and/or meat. Thus, Roskam et al. (2022) [161] investigated the antimethanogenic potential of bromoform-free brown and green seaweeds (*Pelvetia canaliculata*, *Ericaria selaginoides* (formerly *Cystoseira tamariscifolia*), *Bifurcaria bifurcata*, *Fucus vesiculosus*, *Himanthalia elongata*, *Ascophyllum nodosum*, and *Ulva intestinalis*) in vitro. The results showed that only *Fucus vesiculosus* reduced CH_4_%; however, the absolute methane production was not significantly reduced. Notably, it is the bromoform content that effectively disrupts methanogen’s function, while other bromoform-free seaweeds rich in tannins and phenolic compounds are incapable of mitigating methane formation before impairing the overall rumen habitat. 

On the other hand, studies have also provided insights into methane mitigation properties in PUFA-rich microalgae involving a different mode of action. In the study of Mavrommatis et al. (2021) [162], the inclusion of *Schizochytrium* spp. in goats’ diet decreased the abundance of total archaea and methanogens in the rumen particle-associated microbiota. Moreover, in the rumen liquid fraction the *Methanobrevibacter* spp., a dominant archaeon of the hydrogenotrophic pathway was significantly decreased [163]. The mode of action that resulted in the former changes is related to LCPUFAs contained in *Schizochytrium* spp. [164]. More specifically, it has been proposed that double bonds alter the shape of the molecule, such that kinked unsaturated fatty acids disrupt the bacterial lipid bilayer structure, resulting in chemiosmotic issues and imbalances in acyl CoA metabolism [165]. Nevertheless, recent evidence subverts the aforementioned assumptions, indicating that unsaturated fatty acids did not considerably affect bacterial growth of both Gram-negative and Gram-positive strains [166], while unsaturated fatty acids are involved in the prevention of biofilm formation in Gram-positive bacteria, even at very low levels [166]. Hence, rumen bacterial populations could return to a planktonic lifestyle if the biofilm is dispersed, making them prone to abiotic factors. In addition to methanogen suppression by marine fatty acids (EPA, DPA, and DHA), as reflected by their DNA footprint, methane mitigation was observed in an in vitro study [167]. 

## 10. Conclusions 

Microalgae have the potential to revolutionize biotechnology in a number of areas, including feed, nutrition, pharmaceuticals, cosmeceuticals, and biofuels. The biological and chemical diversity of the microalgae has been the source of unique antioxidant molecules with the potential for industrial development as feed nutritional supplements. Microalgal biomass is an attractive alternative to traditional forms of biomass for the production of high value-added antioxidants due to high productivity, the ability to be cultivated on marginal lands, and the potential to utilize carbon dioxide. Since microalgal biomass is still largely unexplored, it represents a rich source for discovery in both academic and industrial sectors. Further research should be performed aiming to assess the potential of microalgae in substituting synthetic antioxidants (e.g., vitamin E) in animal feed since the synthetic ones have been linked with severe concerns for human health [168]. To bridge this scientific gap, targeted experimental trials should be designed in order to validate the equivalent of vitamin E antioxidant activity of specific microalgae in vivo. Additionally, although extensive evidence supports the antimicrobial potency of microalgae, scarce information exists about the in vivo antibacterial, antiprotozoal, and antihelminthic effects of biomolecules present in microalgae. This perspective should not only be investigated under pilot conditions but also should be validated at a commercial level, where the effect of other cofactors (e.g., pathogens, thermal stress, oxidative stress, social stress, welfare issues, etc.) are also concerned.

In addition to the direct effect of microalgae on improving both animals’ health and performance, there are indirect aspects of their usage in livestock. Microalgae are sustainable natural bioresources that do not compete with terrestrial plants for arable land, freshwater, pesticides, fertilizers, and insecticides to grow, and yet they have high productivity [169]. Thus, substituting synthetic feed additives with microalgae could result in positive environmental outcomes in the livestock sector with higher-quality nutritional products [170,171,172,173,174].
